# Bibliometric analysis of hyperpolarization-activated cyclic nucleotide-gated (HCN)channels research (2004-2020)

**DOI:** 10.1080/19336950.2021.2020005

**Published:** 2022-03-02

**Authors:** Chuanxi Tian, Xueping Zhu, Qiuyuan Wang, Tianyi Lv, Siyi Cheng, Daowen Yang

**Affiliations:** a Graduate School of Beijing University of Chinese Medicine, Beijing, China; b Department of Traditional Chinese Medicine for Pulmonary Diseases, China-Japan Friendship Hospital, Beijing, China; c Cardiovascular Department, Guanganmen Hosptial, China Academy of Chinese Medical Sciences, Beijing, China

## Introduction

The HCN channel family comprises of four members (HCN1-4) expressed in the heart and nervous system. The current produced by HCN channels is known as I-h (or I-f or I-q). I-h has also been designated as pacemaker current because it plays a key role in controlling rhythmic activity of cardiac pacemaker cells and spontaneously firing neurons [1].

The diversity of functions that HCN channels perform is partly attributable to differences in their subcellular localization [2]. HCN channels are highly regulated proteins, which respond to different cellular stimuli, they open at hyperpolarizingpotential, carrymixed Na^+^/K^+^ current, and are regulated by cyclic nucleotides [3]. These channels play important roles in modulating cellular excitability, rhythmic activity, dendritic integration, and synaptic transmission. HCN channel functions range from setting resting potential, synaptic normalization, gain control, after-hyperpolarization, setting responses in dendrites, mediating cannabinoid role in neuronal plasticity, to the gating of plasticity [4]. These functions have been implicated in a wide range of diseases, including major depressive disorder, neuropathic pain, and multiple subtypes of epilepsy [4,5.

Among the four known isoforms, HCN1 is the most expressed in the neocortex and hippocampus. Some studies suggest that coordinated changes in protein expression and surface expression of HCN1 serve as the key regulatory mechanisms controlling the function of the endogenous HCN1 protein in cortical neurons [6]. HCN1 might be involved in reduced vagal modulation and possibly in increased cardiac mortality in schizophrenia patients [7].

HCN2 ion channel activity plays a crucial role in the progress of peripheral neuropathic pain (PNP). Some studies suggest that HCN2 contributes to the development of neuropathic pain by inducing spinal LTP via activation of NMDA receptor-mediated CaMKII signaling, decreased HCN2 channel expression attenuates neuropathic pain by inhibiting pro-inflammatory reactions and NF-kappa B activation[8,9]

Like all other HCNs, hHCN3 was inhibited rapidly and reversibly by extracellular cesium and slowly and irreversibly by extracellular applied ZD7288. The human channel was not modulated by intracellular cAMP, a hallmark of the other known HCN channels so the missing response to cAMP distinguishes human HCN3 from both the well cAMP responding HCN subtypes 2 and 4 and the weak responding subtype 1[10]. Upregulation of HCN3 channels in IGL neurons is essential for intrinsic excitability and rhythmic burst firing, and PIP2 serves as a powerful modulator of I-h-dependent properties via an effect on HCN3 channel gating[11].

HCN4 is expressed in brain regions relevant to mood and anxiety disorders including specific thalamic nuclei, the basolateral amygdala, and the midbrain dopamine system[12].

The HCN channel subtype selectivity of the GBP provides a unique tool for investigating HCN4 channel function in the central nervous system. The HCN4 channel is a candidate molecular target for the acute analgesic and anticonvulsant actions of GBP[13].

Some studies establish that HCN4 channel played a preventive role in triggering the bradycardia-induced ventricular arrhythmias and halamic HCN4 channels are crucial for the production of rhythmic intrathalamic oscillations and determine the regular TC oscillatory activity during alert states[14–16].

Bibliometric analysis is a widely used tool to assess the academic status of a specific field. Although HCN channels have been a hotspot of multidisciplinary research for decades, a bibliometric analysis of HCN channels has not been published. Here, we collected scientific publications on HCN channels research in the past 17 years, then used CiteSpace 5.7.R5 and VOSviewer1.6.16 for data analysis and visualization to provide some guidance for further research.

## Data collection

The data search was conducted on 14 June 2021 and collected in one day to avoid any potential bias due to the daily update of the database. The search keywords were as follows: TS = (hyperpolarization-activated cyclic nucleotide-gated cation channel* OR HCN channels), language:(English), and year range: (2004–2021). The data for the analysis were extracted from the Science Citation Index Expanded (SCI-expanded) of Web of Science Core Collection (WoSCC) database. (The data in this research comes from public databases and does not require ethical approval.)

**Figure 1.**
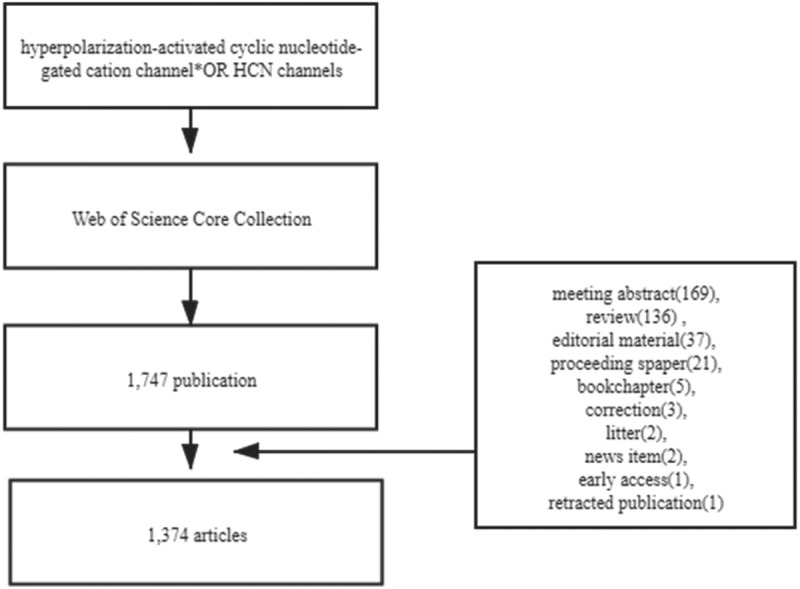
Flow chart of HCN channels researches inclusion.

A total of 1,747 publications were obtained, and the following documents were excluded meeting abstract (169), review (136), editorial material (37), proceedings paper (21), book chapter (5), correction (3), litter (2), news-item (2), early-access (1), retracted-publication (1). In total, 1,374 articles were included. The retrieval strategy is shown in Figure 1. VOSviewer1.6.16 was used to identify top countries, institutions, authors, journals, and co-cited references. The CiteSpace 5.7.R5 was used to analyze keywords and trends.

## General information and annual publication output

**Figure 2.**
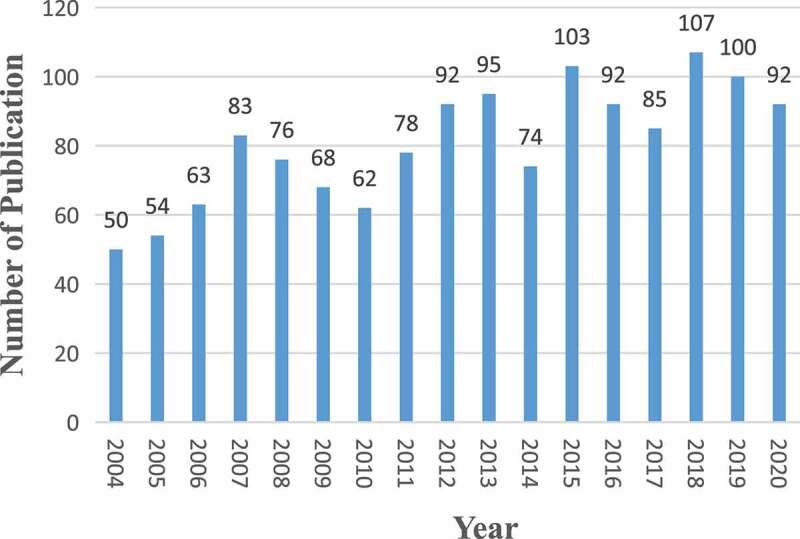
The number of annual publications on channels research from 2004 to 2020.

A total of 1,374 articles about HCN channels were published from 2004 to 2021. To explore the trend of HCN channels research, we describe the number of articles per year in the form of a histogram. As is shown in Figure 2, the number of publications on HCN channels research increased gradually since 2004, reaching a peak in 2018. The average annual number of publications was 80.8.

## Active countries and institutions

**Figure 3.**
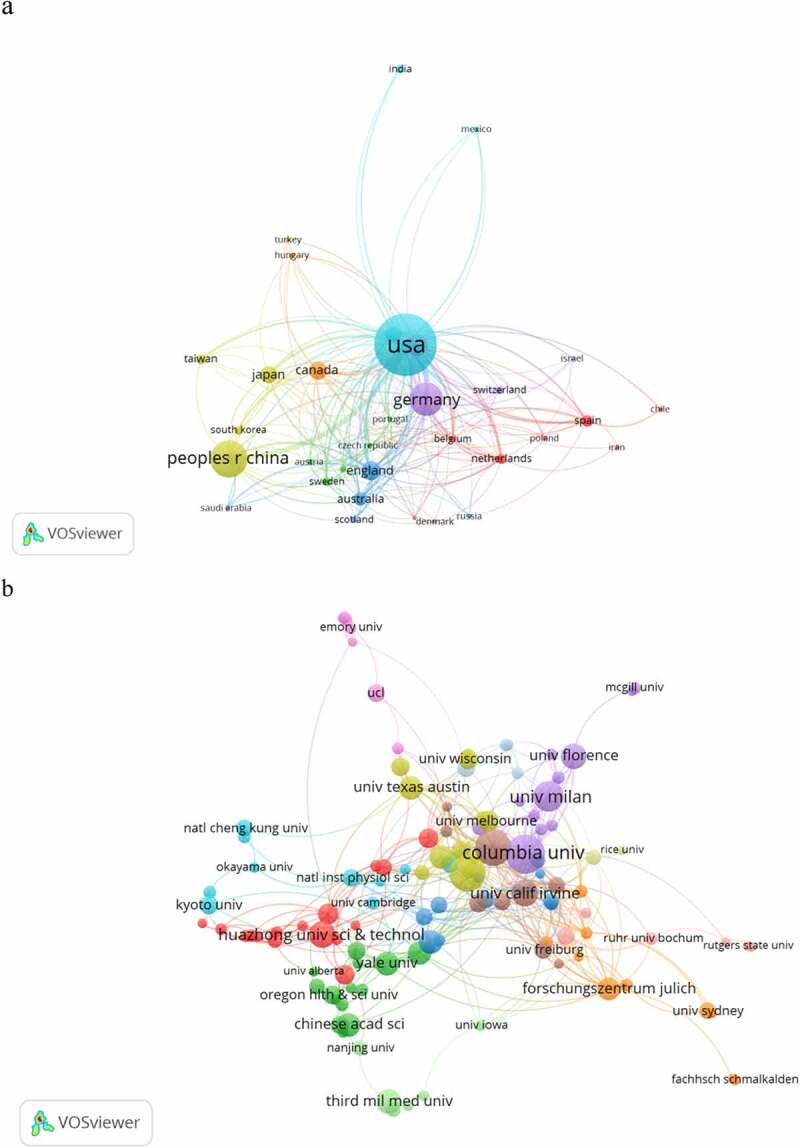
The analysis of countries and institutions. (a). The network of countries/territories engaged in HCN channels research; (b). The network of institution engaged in HCN channels research.

The co-occurrence map provides valuable information and helps researchers to identify the cooperative relationship [17]. Table 1 lists the top 10 countries and institutions that contributed to HCN channels research. Countries and institutions co-occurrence networks are shown in Figure 3.

**Table 1.** The top 10 **c**ountries and institutions contributed to Publication on HCN channels research

Rank	Country /Territory	Frequency	Institution	Frequency
1	USA	558	Columbia University	43
2	Peoples R China	251	University of Washington	39
3	Germany	212	Northwestern University	38
4	Italy	105	University of Milan	29
5	England	86	University of California, Irvine	23
6	Canada	78	Huazhong University of Science and Technology	23
7	Japan	77	University of Florence	22
8	France	69	Third Military Medical University	21
9	Australia	54	University of Texas at Austin	20
10	Spain	40	University of Munich	20

The 1,374 articles on HCN channels research were published by more than 1,226 research institutions in 62 countries/territories. The USA, Peoples R China, Germany, Italy, and England were the top five productive countries/territories (Table 1). As is shown in Figure 3, Columbia University produced the highest number of publications on HCN channels research (43), followed by the University of Washington (39) and Northwestern University (38).

## Active journals

The 1,374 articles were published in 401 journals. Table 2 lists the top 10 journals that published articles on HCN channels research. The Journal of Neuroscience had the highest number at 70 (17.45%), followed by Journal of Biological Chemistry and Journal of Neurophysiology, both with 45 papers (3.27%).

**Table 2.** The top 10 journals that have published articles on HCN channels research

Rank	Journal	Frequency	IF 2019	Country Affiliation
1	Journal of Neuroscience	70(17.45%)	5.674	UNITED STATES
2	Journal of Biological Chemistry	45 (3.27%)	4.238	UNITED STATES
3	Journal of Neurophysiology	45 (3.27%)	2.234	UNITED STATES
4	Journal of Physiology-London	43(3.13%)	4.547	UNITED STATES
5	Journal of Physical Chemistry A	41(2.98%)	2.600	UNITED STATES
6	PLoS One	40(2.91%)	2.740	UNITED STATES
7	Journal of General Physiology	38(2.77%)	3.628	UNITED STATES
8	Neuroscience	28(2.04%)	3.056	ENGLAND
9	Proceedings of the National Academy of Sciences of the United States of America	23(1.67%)	9.412	UNITED STATES
10	European Journal of Neuroscience University of Munich	23(1.67%)	3.115	UNITED STATES

## Active authors

**Figure 4.**
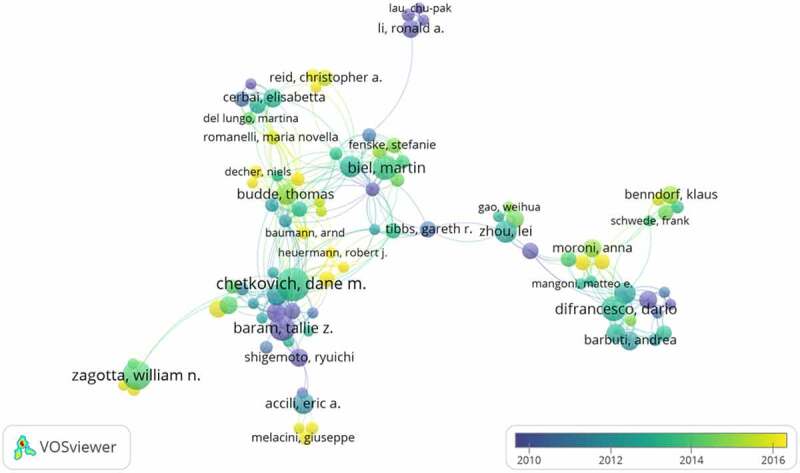
The network of authors contributed to HCN channels research.

Approximately 5,918 authors contributed over 1,374 articles related to HCN channels research. Figure 4 indicates the cooperation among authors, and the top 10 active authors are listed in Table 3. Chetkovich, Dane M, who mainly concentrated on the TRIP8b-HCN interaction and the role of HCN channels and I-h in behavior and disease, was the most prolific author in terms of publications on HCN channels (24 papers) [18–20]. Followed by Zagotta, William N. (20 papers) whose research mainly focused on Structural Determinants of the Hyperpolarization-Dependent Gating and Electromechanical coupling mechanism of HCN Channels [21,22], and Baram, Tallie Z. (17 papers) with publications mainly on the aspect of abnormalities in the HCN2 isoform and the dynamics of HCN channel trafficking in hippocampal neurons and HCN Channels in Epilepsy.

**Table 3.** The top 10 active authors in HCN channels research

Rank	Author	Frequency
1	**Chetkovich, Dane M**	24
2	**Zagotta, William N.**	20
3	**Baram, Tallie Z.**	17
4	Difrancesco, Dario	16
5	Biel, Martin	16
6	Zhou, Lei	14
7	Bucchi, Annalisa	13
8	Ludwig, Andreas	13
9	Accili, Eric A.	13
10	Budde, Thomas	12

## Co-cited references

**Figure 5.**
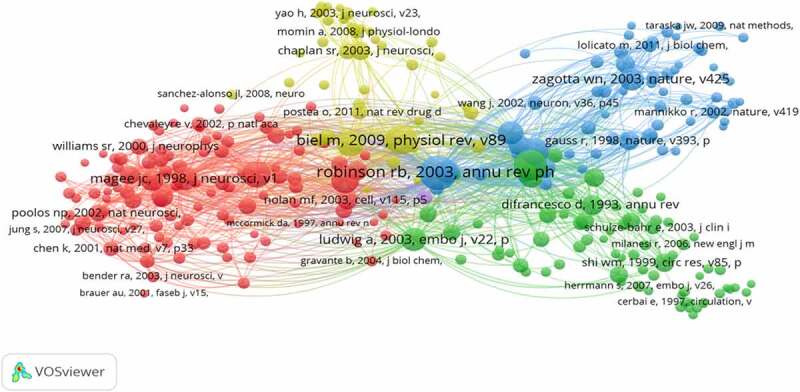
The analysis of Co-cited references: Co-citation network of references from publications on HCN channels research.

The 1,374 articles about HCN channels were visualized and analyzed through VOSviewer1.6.16. The co-cited reference network (Figure 5) presents the references with higher centrality and citation counts. The top 10 highly co-cited references are summarized in Table 4.

**Table 4.** The top 10 highly co-cited (CR) in HCN channels research

Rank	Frequency	Author	Year	Source	Co-cited Reference
1	349	**Robinson RB**	2003	Annual Review of Physiology	Hyperpolarization-activated cation currents: from molecules to physiological function.
2	276	**Ludwig A**	1998	Nature	A family of hyperpolarization-activated mammalian cation channels.
3	268	**Biel M**	2009	Physiological Reviews	Hyperpolarization-Activated Cation Channels: From Genes to Function.
4	210	**Santoro B**	1998	Cell	Identification of a gene encoding a hyperpolarization-activated pacemaker channel of brain.
5	207	**Notomi T**	2004	Journal of Comparative Neurology	Immunohistochemical localization of I-h channel subunits, HCN1-4, in the rat brain
6	198	**Pape HC**	1996	Annual Review of Physiology	Queer current and pacemaker: the hyperpolarization-activated cation current in neurons.
7	193	**Santoro B**	2000	The Journal of Neuroscience	Molecular and functional heterogeneity of hyperpolarization-activated pacemaker channels in the mouse CNS.
8	167	Ludwig A	2003	The EMBO Journal	Absence epilepsy and sinus dysrhythmia in mice lacking the pacemaker channel HCN2.
9	167	**Wainger BJ**	2001	Nature	Molecular mechanism of cAMP modulation of HCN pacemaker channels.
10	166	**Magee JC**	1998	The Journal of Neuroscience	Dendritic hyperpolarization-activated currents modify the integrative properties of hippocampal CA1 pyramidal neurons.

The first highly co-cited article was “Hyperpolarization-activated cation currents: from molecules to physiological function” (349 citation rates), in which Robinson RB reviewed and summarized the Hyperpolarization-activated cation currents from molecules to physiological function [23]. Biel M reviewed recent insights into the structure, function, and cellular regulation of HCN channels [24]. Pape HC reviewed the hyperpolarization-activated cation current in neurons [25]. These reviews provide a theoretical basis for the study of HCN channels.

Two co-cited references were published in Nature: In 1998, Ludwig A reported the molecular cloning and functional expression of the gene encoding a hyperpolarization-activated cation channel (HAC1) that is present in the brain and heart, and indicated the existence of a family of hyperpolarization-activated cation channels through HAC2 and HAC3 that are specifically expressed in the brain [26]. In 2001, Wainger BJ demonstrated that the differences in activation gating and extent of cAMP modulation between HCN1 and HCN2 isoforms result largely from differences in the efficacy of CNBD inhibition[27].

Two co-cited references were published in The Journal of Neuroscience. In 1998, Magee JC confirmed that Ih acts to dampen dendritic excitability pacemaker channels in the mouse CNS [28]. Santoro B researched the Molecular and functional heterogeneity of hyperpolarization-activated, he also cloned mBCNG-1, a gene from the mouse brain and proposed it to be a candidate gene for pacemaker channels [29]. Notomi T examined precise immunohistochemical localization of four HCNs (HCN1-4) in the rat brain, and his study supported previous electrophysiological findings and further suggested unexpected roles of Ih channels in the brain [30].

Ludwig A, whose experiments showed that HCN2-deficient mice exhibit spontaneous absence of seizures. These articles laid the foundation for studying the structure, function, and cellular regulation of HCN channels.

## Research area analysis

**Figure 6.**
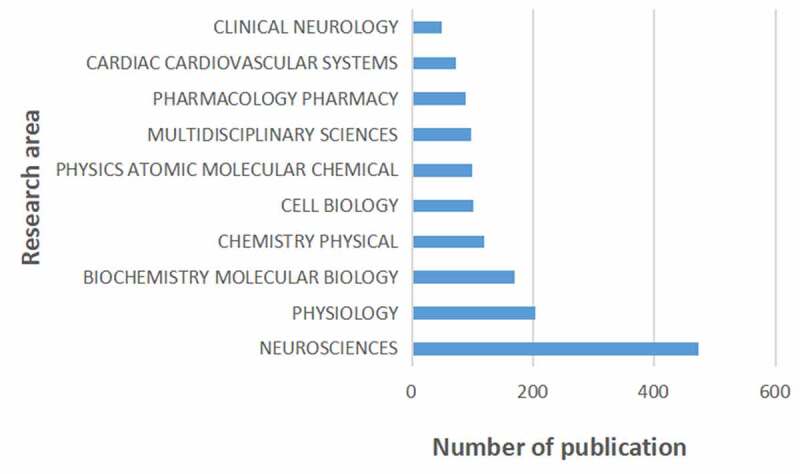
The 10 research areas on HCN channels research.

Figure 6 shows the top 10 research areas that appeared in publications related to HCN channels research from 2004 to 2020. Neurosciences, Physiology, Biochemistry Molecular Biology are the top three areas of HCN channels.

## Keywords co-occurrence and burst

Keyword co-occurrence analysis provides a reasonable description of research hotspots, and burst keywords can represent research frontiers over a period of time [31].

**Figure 7.**
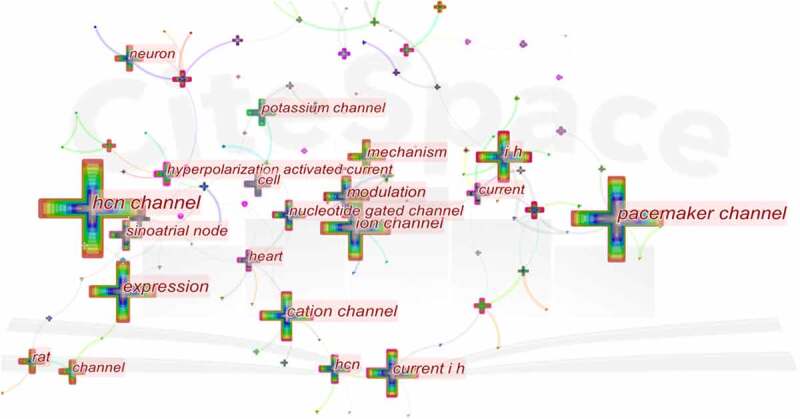
The analysis of keywords in HCN channels research.

CiteSpace 5.7.R5 were used to construct acknowledge map of keyword co-occurrence (Figure 7), and the top 25 keywords in HCN channel research articles from 2004 to 2021 were identified according to frequency (Table 5). The top five keywords were “hcn channel,” “pacemaker channel,” “expression,” “i h,” “ion channel.” Therefore, research hotspots can be summarized in the following aspects:

**Table 5.** Top 20 keywords in HCN channels research

Rank	Keywords	Frequency	Rank	Keywords	Frequency
1	HCN channel	374	14	neuron	84
2	pacemaker channel	263	15	rat	83
3	expression	172	16	channel	83
4	i h	157	17	potassium channel	78
5	ion channel	157	18	current	77
6	cation channel	127	19	excitablity	73
7	modulation	112	20	hyperpolarization activated current	72
8	current i h	112	21	heart	67
9	HCN	98	22	activated cation channel	62
10	nucleotide gated channel	94	23	pacemaker	61
11	sinoatrial node	88	24	neuropathic pain	59
12	mechanism	86	25	camp	57
13	cell	85

### Cardiac and neuronal HCN channelopathies

Hyperpolarization-activated cyclic nucleotide-gated (HCN) channels are expressed in the heart and in the central and peripheral nervous systems. In the voltage range of activation, HCN channels carry an inward current mediated by Na^+^ and K^+^, termed I-f in the heart and I-h in neurons. The number of both experimental models and clinical studies is growing, indicating that the alteration of HCN ion channels is implicated in the pathogenesis of different diseases, especially in cardiac and neurological system [32].

### Isoforms of HCN channels

Among the four isoforms of the HCN channel, HCN2 and HCN4 are predominant in the heart, with HCN4 prevailing in the SAN and HCN2 in the ventricle [32]. HCN3 makes some contribution to excitability, particularly in medium-sized neurons, although it has no major influence on acute or neuropathic pain processing [33].

### Gating mechanism of hyperpolarization-activated HCN channels

Hyperpolarization-activated cyclic nucleotide-gated (HCN) channels are essential for rhythmic activity in the heart and brain, and mutations in HCN channels are linked to heart arrhythmia and epilepsy. HCN channels belong to the family of voltage-gated K^+^ (Kv) channels. A study indicated that the small differences in the energies of the closed and open states during different interactions between the voltage sensor and the pore in the different channels determines the opening of HCN channels by hyperpolarizations or depolarizations [34].

Keywords were identified and analyzed using a strong citation burst (Table 6) to explore the frontiers of research. As shown in Table 6, the red line indicates the period of time during which the burst keyword appears. The keywords that had strong bursts after 2015 were “neuropathic pain” (2015–2020), “ivabradine” (2017–2020), “structural basis” (2017–2020), “channel” (2017–2020), “rat” (2017–2020). The two research frontiers of HCN channels were as follows

**Table 6.** Top 25 keywords with strongest citation burst

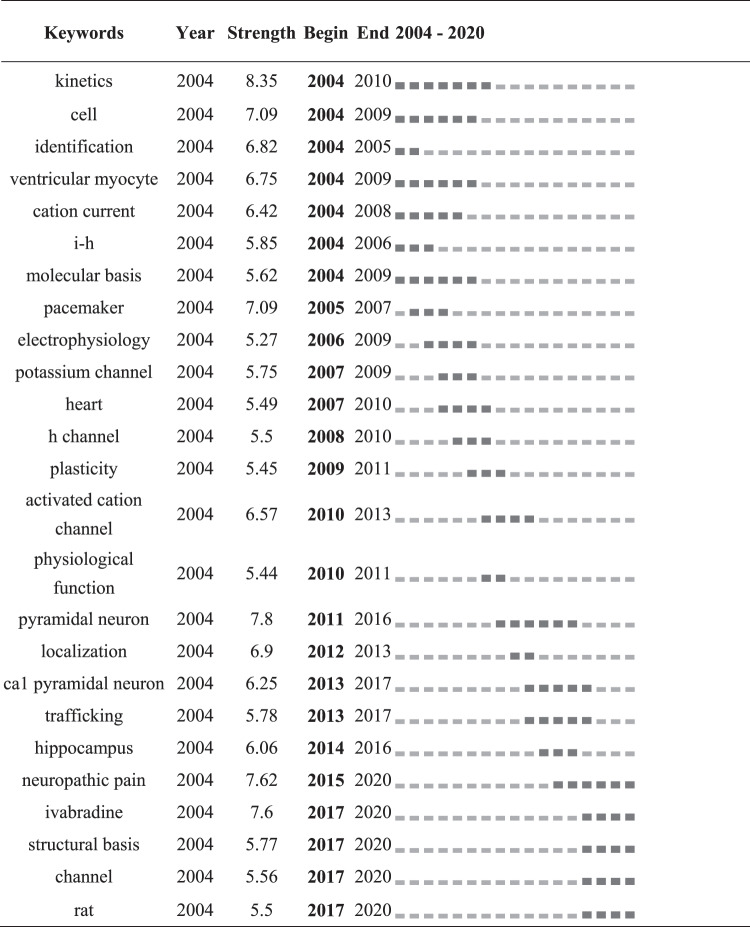

### Neuropathic pain

Neuropathic pain is a disease with global burden. Its symptoms include spontaneous and stimulus-evoked painful sensations [35]. Studies have documented the pain-attenuating actions of selective HCN inhibitors, such as ivabradine and ZD7288. Moreover, certain drugs with additional HCN-blocking activities have also shown pain-attenuating actions in different pain models. Hyperpolarization-activated and cyclic nucleotide-gated channel proteins are viewed as emerging new targets in neuropathic pain.

### Ivabradine

Ivabradine is a hyperpolarization-activated cyclic nucleotide-gated channel inhibitor and a specific bradycardic agent used in coronary artery disease and heart failure, lowering heart rate through inhibition of sinoatrial nodal HCN-channels [36].

In the sinoatrial node (SAN) HCN4 is the target of ivabradine. Ivabradine is the only drug, which specifically blocks I-f and the first HCN channel inhibitor being clinically approved in 2005 for the treatment of chronic stable angina pectoris and heart failure [37,38].

A new study found that systematic administration of ivabradine blocks spontaneous absence seizures, which indicated the potential of this class of drugs as a novel therapeutic avenue for Ass [39].

## Conclusion

Based on the WoSCC database, bibliometric and visual analysis were conducted to study the characteristics of HCN channels research from 2004 to 2021. Since 2004, the average annual number of publications was 80.8 per year. The top three hot spots of HCN channels were “Cardiac and neuronal HCN channelopathies,” “Isoforms of HCN channels,” “Gating mechanism of hyperpolarization-activated HCN channels.” The two research frontiers of HCN channels were “Neuropathic pain” and “Ivabradine.” Bibliometric analysis of the literature on HCN channels may help researchers to identify cooperations, find hot spots, and predict the future of HCN channels research.
